# Hierarchical channel morphology in O-rings after two cycling exposures to 70 MPa hydrogen gas: a case study of sealing failure

**DOI:** 10.1038/s41598-024-55101-w

**Published:** 2024-03-04

**Authors:** Chang Hoon Lee, Jae Kap Jung, Kyung Sook Kim, Chang Jong Kim

**Affiliations:** 1 Department of Biochemical Engineering, Chosun University, Chosundae-5-gil, Dong-gu, Gwangju, 61452 Republic of Korea; 2 Hydrogen Energy Materials Research Team, Korea Research Institute of Standards and Science, Daejeon, 34113 Republic of Korea; 3 Department of Biomedical Engineering, College of Medicine, Kyung Hee University, Seoul, 02447 Republic of Korea; 4 LG Chem Europe GmbH, Adolph-Prior-Straße 16, 65936 Frankfurt am Main, Germany

**Keywords:** Engineering, Materials science

## Abstract

This study investigates the impact of high-pressure hydrogen gas exposure on the structural and morphological characteristics of O-ring materials. O-ring specimens undergo two cycles of sealing under 70 MPa hydrogen gas, and their resulting variations are examined using advanced characterization techniques, including powder X-ray diffraction (PXRD), small-angle X-ray scattering (SAXS), scanning electron microscopy (SEM) and atomic force microscopy (AFM). Our findings reveal that the lattice parameters of the O-ring material show no significant changes when exposed to 70 MPa hydrogen gas. However, in the micrometre range, the formation of a hierarchical channel morphology becomes evident. This morphology is accompanied by the separation of carbon black filler from the rubber matrix, contributing to mechanical weakening of the O-ring. These observations can be attributed to the pressure gradient that develops between the inner and outer radii of the O-ring, resulting from compression forces acting perpendicularly to the radial direction due to clamp locking.

## Introduction

The urgency of the climate crisis has surpassed initial expectations, presenting a global challenge accelerating at an unprecedented pace. In response, human efforts are resolutely focused on achieving carbon neutrality through diverse initiatives^1^. A critical step in this pursuit is reducing carbon dioxide emissions^2^ from internal combustion engines^3^, with a notable strategy involving the shift to electric or hydrogen fuel cell vehicles^4,5^. While the transition to electric vehicles progresses smoothly, the challenge of reducing charging times remains a significant obstacle.

Hydrogen fuel cell vehicles^4,6^ offer environmental benefits, using eco-friendly hydrogen as fuel, contributing to air purification, and generating only water as a byproduct. However, ongoing debates persist about psychological resistance to safety issues during the expansion of hydrogen infrastructure^7^. Concerns also linger regarding the environmental impact of hydrogen production and the efficacy of carbon dioxide reduction. Addressing these technological and psychological challenges is crucial for implementing these solutions across terrestrial, maritime, and aerial transportation.

Currently, hydrogen fuel cell vehicles in the market use hydrogen dispensers at gas stations, storing hydrogen at a pressure of 90 MPa. The type-4 pressure vessel in these vehicles has a maximum pressure of 70 MPa, with O-rings primarily composed of rubber composites serving as the primary means to seal the hydrogen gas pressure^8,9^. The O-ring consists of polymer matrices, including cross-linked rubbers or amorphous fluoroelastomers, along with additives like carbon black and plasticizers. When an O-ring seals H_2_ gas, its effectiveness degrades over time due to cyclic pressure and temperature variations during the pressurization and depressurization process^10–12^. Eventually, the O-ring fails to maintain a proper seal^9,13,14^.

The mechanical damage to rubber O-rings caused by hydrogen gas includes blister fracture, overflow fracture, and buckling fracture^15^. At the microscopic level, hydrogen gas dissolved in polymer materials can form micro-sized clusters, known as hydrogen gas clustering. During depressurization, these clusters can coalesce to create hydrogen gas bubbles, gradually expanding over time after depressurization^9,16,17^. This expansion exerts stress, serving as blister initiation points. Research supports evidence of bubble formation and expansion, with the potential for cutting polymer chains during blister formation confirmed through studies introducing side-chain molecules with fluorescent characteristics^18^.

Ongoing studies aim to comprehensively understand the mechanical and chemical changes^9,19^ during hydrogen gas depressurization. To address this issue, the development of polymeric materials that exhibit long-term durability and the exploration of new processing routes for manufacturing mechanically superior polymers and composites are crucial^15^.

Previous research efforts have primarily focused on investigating rubber composites exposed to ambient H_2_ pressure^20–25^. However, in practical engineering applications, the O-ring seal encounters an outward H_2_ pressure gradient, spanning from high pressure at the inner radius to low pressure at the outer radius. Additionally, the O-ring is subjected to mechanical confinement due to normal uniaxial forces acting perpendicular to the radial direction^26^. Consequently, conducting studies that specifically examine the behaviour and deterioration mechanisms of polymeric materials in the context of O-ring usage in engineering practice is crucial.

## Results and discussion

**Figure 1:**
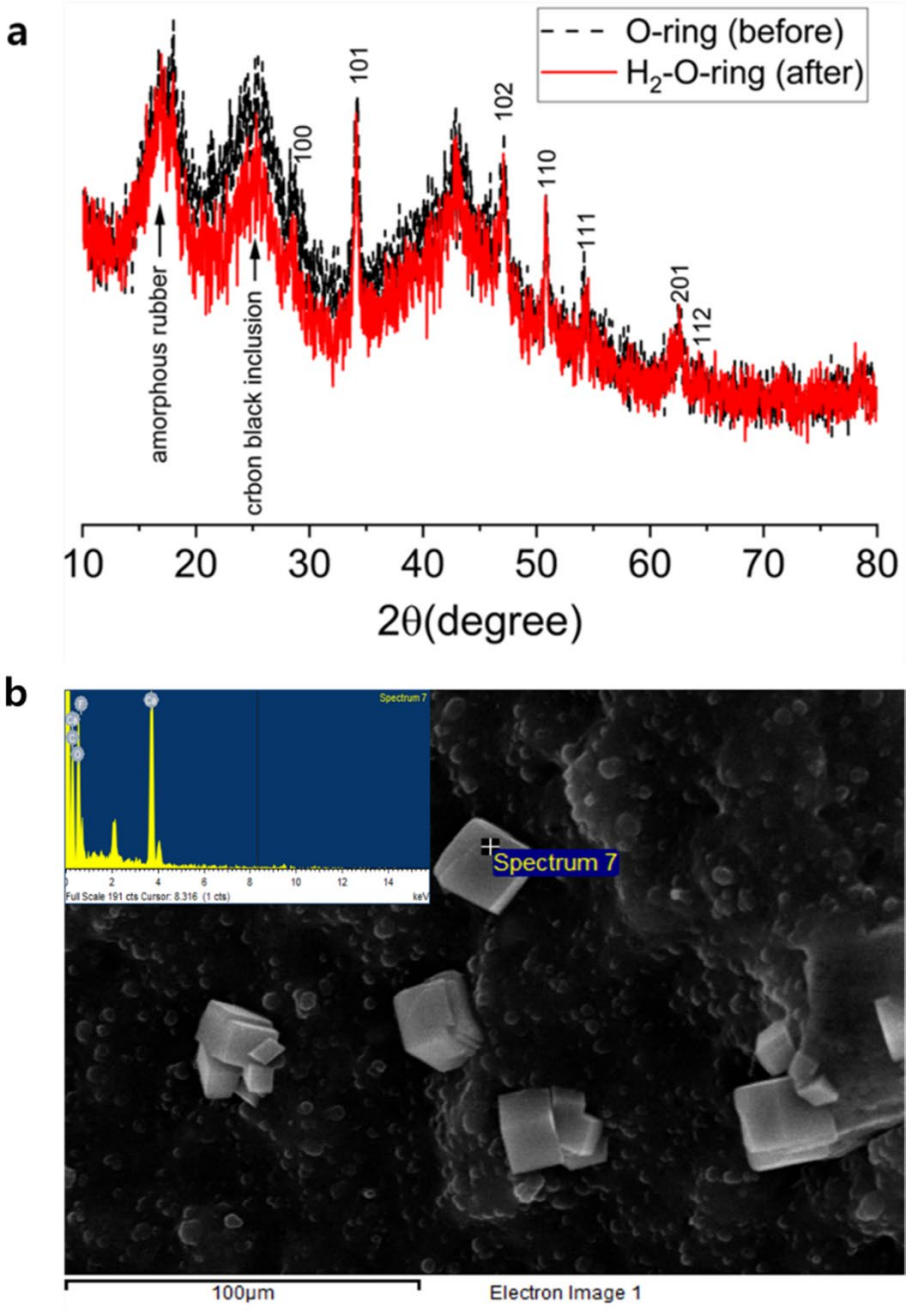
(**a**) PXRD data obtained for the O-ring before and after exposure to H_2_ at a pressure of 70 MPa. The names displayed at the peak positions represent the origin of each peak, while the Arabic sequences at the peak positions indicate the Miller indices for the peaks derived from Ca(OH)_2_. (**b**) SEM image of the O-ring showing a black background representing the rubber composite with the carbon black filler and white cube-like crystals indicating Ca(OH)_2._ The inset figure displays the EDS spectrum of the Ca(OH)_2_ crystal located at the uppermost position, labeled as spectrum.

PXRD analysis was performed to investigate any changes in the lattice spacing of the polymer matrix after exposure to H_2_ at a pressure of 70 MPa. Figure 1 presents the PXRD results obtained for the O-ring specimens before and after exposure to H_2_.

The XRD patterns exhibit three broad peaks at approximately 2θ ≈ 17°, 25°, and 42°, as well as seven sharp peaks at 2θ values of 28°, 34°, 47°, 51°, 54°, 63°, and 65°. Among the broad peaks, the one at 2θ ≈ 25° is attributed to the presence of carbon black inclusions^27–29^, while the peak at 2θ ≈ 17° corresponds to the conventionally observed amorphous phase for polymers^30^. The peak at 2θ ≈ 42° is likely a second harmonic of the 2θ ≈ 25° peak. The seven sharp peaks observed may be indicative of an unknown crystalline phase within the O-ring. However, since the O-ring is reported to be an FKM copolymer consisting of VDF/HFP = 76.1/23.9 mol% (VDF: vinylidene fluoride (CF_2_=CH_2_); HFP: hexafluoropropylene (CF_2_=CF(CF_3_)))^31^, the presence of a crystalline phase is not expected. Therefore, the seven sharp peaks are attributed to another inorganic material rather than the polymer itself. In our case, the peaks are well matched with the Miller indices of Ca(OH)_2_^32,33^ in ICDD(International Centre for Diffraction Data) card number of 00-074-0733, and this is further confirmed by the FE-SEM image^32^ shown in the inset picture in Fig. 1. In result, the XRD pattern analysis identified three primary influencing factors. Firstly, the sharp peaks were attributed to Ca(OH)_2_, supported by provided Miller indices. The second peak (2θ ≈ 25°) corresponds to carbon black inclusion within the sample, while the third peak (2θ ≈ 17°) is associated with entangled rubber polymer chains. Apart from these peaks, no significant variations are observed in the 2θ positions and intensities after exposure to H_2_, indicating no change in the lattice parameters of the polymer matrix^34^.

**Figure 2:**
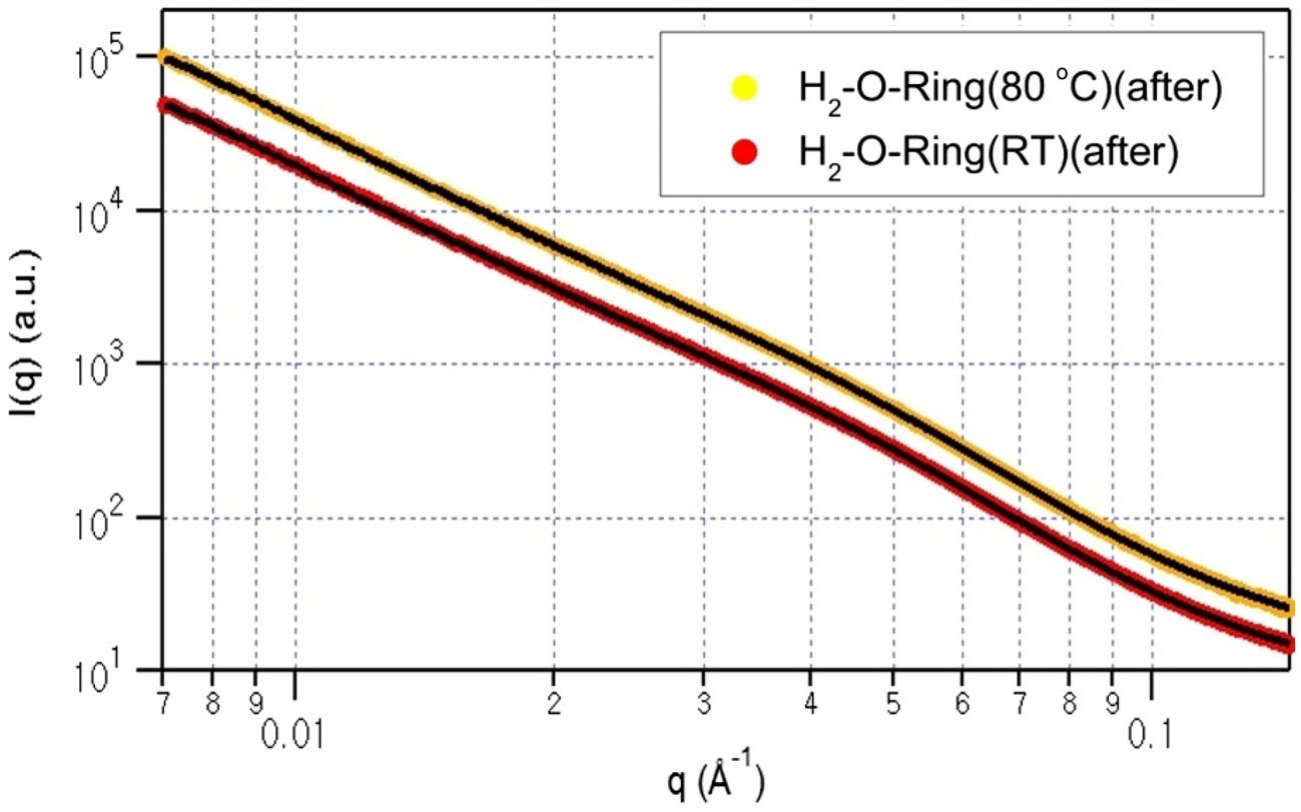
SAXS data for the O-ring collected at room temperature (upper) and 80 °C (lower) before being exposed to H_2_ at a pressure of 70 MPa. The black solid lines represent the fits to Eq. (1).

**Figure 3:**
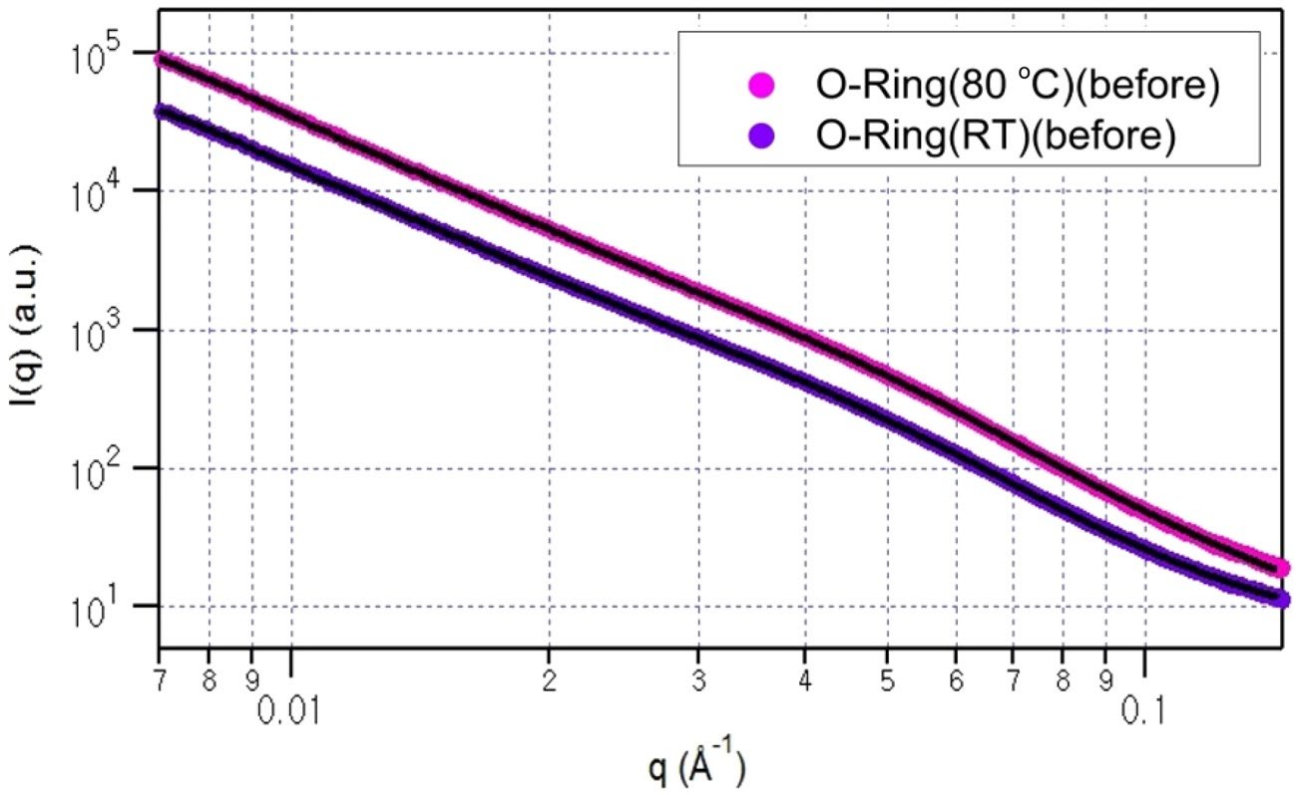
SAXS data for the O-ring collected at room temperature (upper) and 80 °C (lower) after being exposed to H_2_ at a pressure of 70 MPa. The black solid lines represent the fits to Eq. (1).

Subsequently, we conducted SAXS measurements to meticulously monitor and analyse the structural variations covering from a few nanometers to a hundred nanometer. Figures 2 and 3 depict the SAXS data obtained at room temperature and 80 °C before and after, respectively, exposure to H_2_ at a pressure of 70 MPa. Each SAXS profile exhibits the typical scattering patterns from aggregates. For data analysis, the following equations were applied^35–39^:

1 $${\text{I}}\left({\text{q}}\right)=\mathrm{k }\cdot \left\langle {\left|{\text{F}}\left({\text{q}}\right)\right|}^{2}\right\rangle +{I}_{agg} \left(q\right)+{I}_{b}$$

2 $${\text{F}}\left({\text{q}}\right)= \frac{{\text{sin}}\left({\text{q}}{R}_{c}\right)-{\text{qRcos}}\left({\text{q}}{R}_{c}\right)}{{\left({\text{q}}{R}_{c}\right)}^{3}}$$

3 $${{\text{I}}}_{\mathrm{agg }\left({\text{q}}\right)}={\text{G}}\cdot {\text{exp}}\left[-\frac{{Q}^{2}{R}_{g}^{2}}{3}\right]+B\cdot {\left\{\frac{{\left[{\text{erf}}\left(\frac{q{R}_{g}}{\sqrt{6}}\right)\right]}^{3}}{q}\right\}}^{p}$$

where F(q) represents the form factor of a solid sphere, I_agg_(q) is a structure factor used to describe an aggregate, and I_b_ represents the experimental background. The R_c_ term in the form factor is the core radius, and R_g_ in the aggregate structure factor is the radius of gyration of an aggregate. G and B are constants, and P is a power law exponent used to describe the dimensionality of an aggregate (e.g., P = 1 for a rod, P = 2 for a disk, P = 4 for a sphere). Additionally, the form factor F(q) is convoluted with the Schulz size distribution to describe the size distribution of the carbon black (CB) particles, considering the observed presence of CB inclusion in the SEM image in Fig. 1b.

In Figs. 2 and 3, the experimental data are presented alongside the data fits (black solid lines) generated using Eq. (1). To analyse the experimental data obtained before and after exposure to H_2_, the fit parameters derived from the room-temperature measurements, namely, the core radius and size distribution of the core radius, were employed for the analysis of the data collected at 80 °C. This approach ensures a reliable fit by fixing the core radius and size distribution parameters based on the room-temperature measurements while allowing the other parameters (R_g_, P, G and B) to vary during the fitting process for the 80 °C measurements.

**Figure 4:**
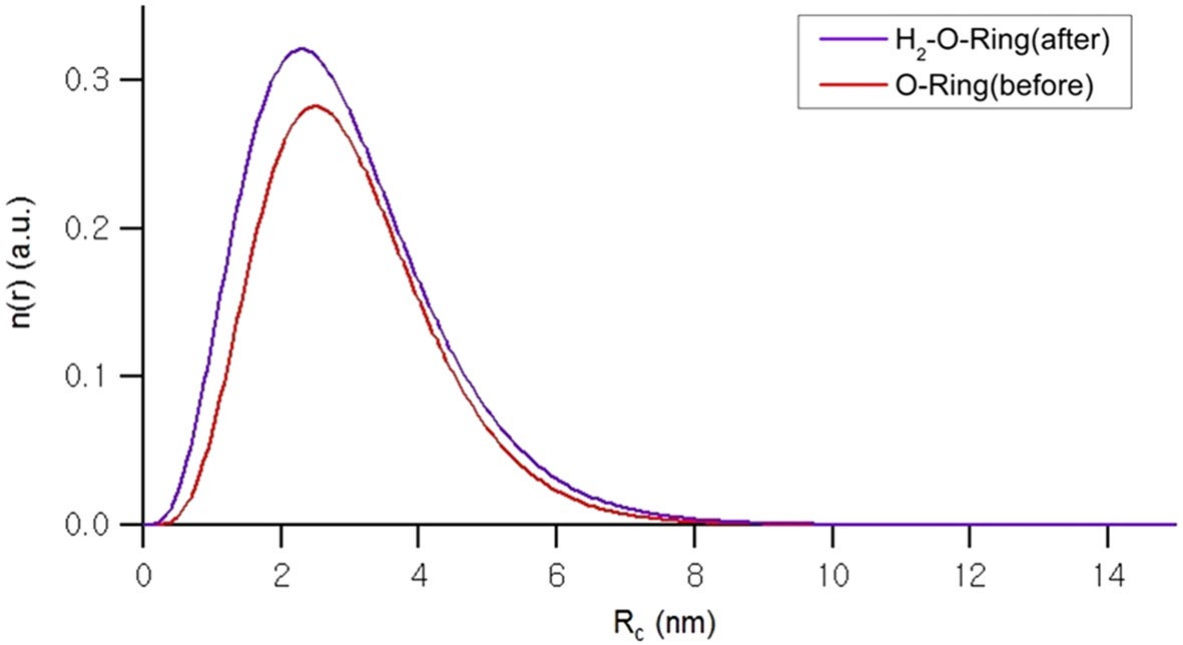
Size distribution of the core radii at room temperature for O-ring specimens before (red) and after (blue) exposure to 70 MPa hydrogen gas. The y-axis represents the size distribution, denoted n(r), and is presented in arbitrary units.

Figure 4 illustrates the resulting size distributions, and the corresponding fit parameters can be found in Table 1. In the room-temperature measurements, the core radii (R_c_) of the H_2_-exposed O-ring is clearly slightly smaller than that of the fresh O-ring. However, the polydispersity index (p.d.i.), as shown in Fig. 4 and Table 1, increases after exposure to H_2_. Furthermore, the radius of gyration (R_g_) of the H_2_-exposed O-ring, which represents the size of the average aggregate, is approximately 1 nm smaller than that of the fresh O-ring. This suggests that the size of CB aggregates decreases after exposure to H_2_. The dimensionality of the aggregates, represented by P, shows a slight increase from 3.02 to 3.05, indicating a tendency towards a more spherical shape after exposure to H_2_. Similar trends are observed in the data obtained at 80 °C.

**Table 1:** Fit parameters obtained using Eq. (1) for the samples.

Sample	R_c_ (nm)	p.d.i.	R_g_ (nm)	P
O-ring (RT) (before)	3.02 ± 0.04	0.42	31.83 ± 0.38	3.02
O-ring (80 °C) (before)	3.02 (fixed)	0.42 (fixed)	31.04 ± 0.30	3.03
H_2_-exposed O-ring (RT) (after)	2.93 ± 0.05	0.47	30.31 ± 0.34	3.05
H_2_-exposed O-ring (80 °C) (after)	2.93 (fixed)	0.47 (fixed)	30.28 ± 0.32	3.11

**Figure 5:**
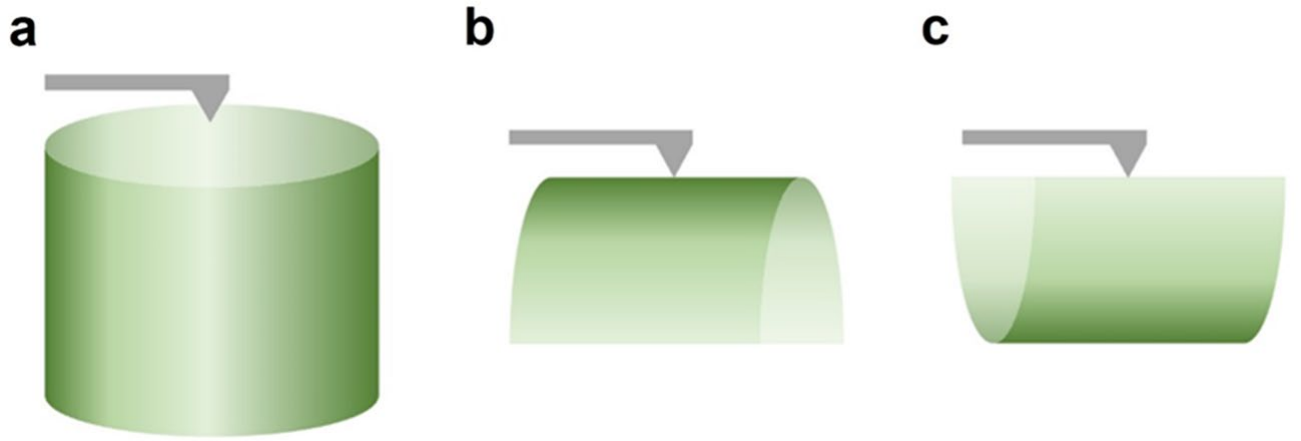
Three different surface areas used for measuring the surface roughness: (**a**) cross-sectional cutting surface, (**b**) outer-radius surface and (**c**) slice cutting surface.

AFM was employed to investigate the morphological changes at the nanometre scale in the O-ring after being exposed twice to hydrogen at a pressure of 70 MPa. AFM images were acquired from three distinct surfaces, as illustrated in Fig. 5.

**Figure 6:**
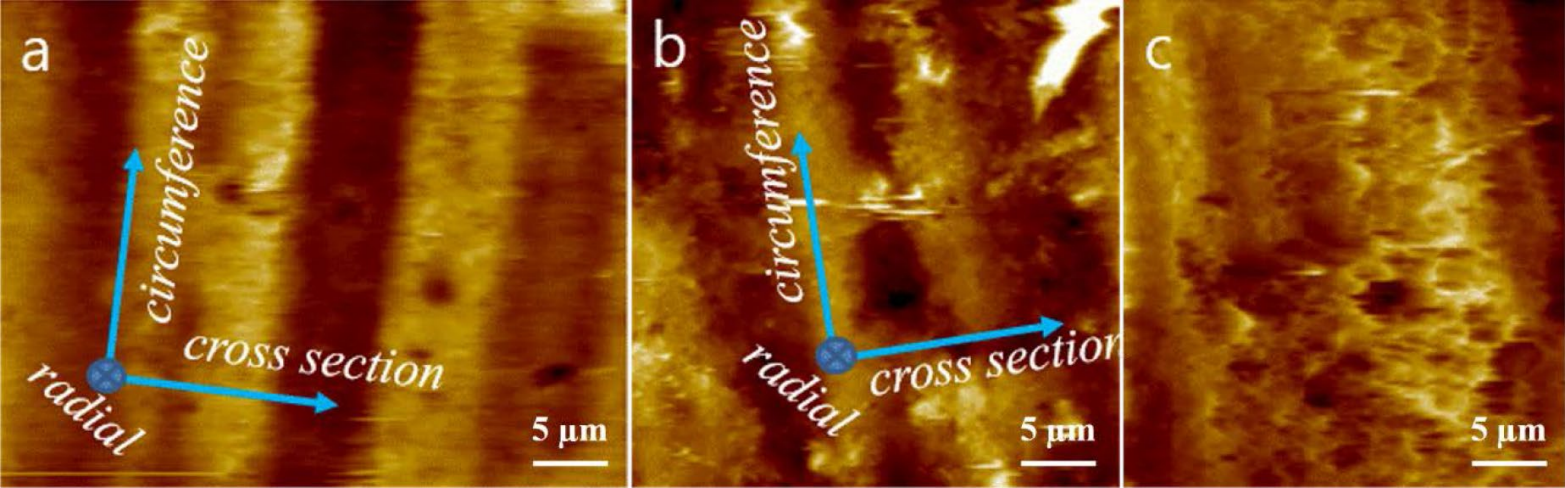
AFM images captured on the outer-radius surface of the O-ring: (**a**) Image taken before exposure to H_2_ at a pressure of 70 MPa. (**b**) Image taken after two exposures to H_2_ at a pressure of 70 MPa. (**c**) Representative image displaying the rupture of the O-ring composite caused by the outburst of H_2_ gas along the radial direction of the O-ring. The illustration positioned in the top-left corner of (**a**) indicates the surface area scanned by the AFM tip.

Figure 6 displays the AFM images obtained from the outer-radius surface of the O-ring. Before exposure to H_2_ (Fig. 6a), a periodic modulation of finite height and width is observed in the cross-sectional plane direction. However, this periodic modulation becomes blurred and merged after exposure to H_2_ (Fig. 6b). Additionally, the morphological roughness, indicated by the colour contrast (white, gold, brown and black), becomes more pronounced and defined. The lighter and darker colours correspond to higher and lower topographies, respectively.

In particular, the areas corresponding to white and black colours are expanded. These phenomena can be explained by considering both the compression stress exerted in the upward and downward directions of the cross-section and the overflow effect due to the H_2_ pressure gradient in the radial direction. The compression stress normal to the radial plane causes the O-ring to strain into an ellipsoidal form, resulting in a smaller distance for the periodic circumferential morphology. At the same time, H_2_ molecules are expected to undergo sequential processes of absorption, diffusion, and permeation through the O-ring, as a hydrogen pressure gradient is established in the inner to outer radial direction. These processes are known to induce swelling, overflow, and fracture of the O-ring composites. To facilitate the merging of the periodic circumferential morphology, narrowing of the periodic morphology due to compression stress and outward displacement of a part of the O-ring composites induced by the pressure gradient must occur simultaneously.

Figure 6c effectively illustrates this outward displacement phenomenon. The region around the black-coloured hole located immediately below the centre of the image protrudes out, forming a bundle of cusps in the outer radial direction, indicating that H_2_ gas burst out through the hole. Naturally, a circular morphology should be observed beneath the hole when the O-ring specimen is imaged on a slice cutting surface.

**Figure 7:**
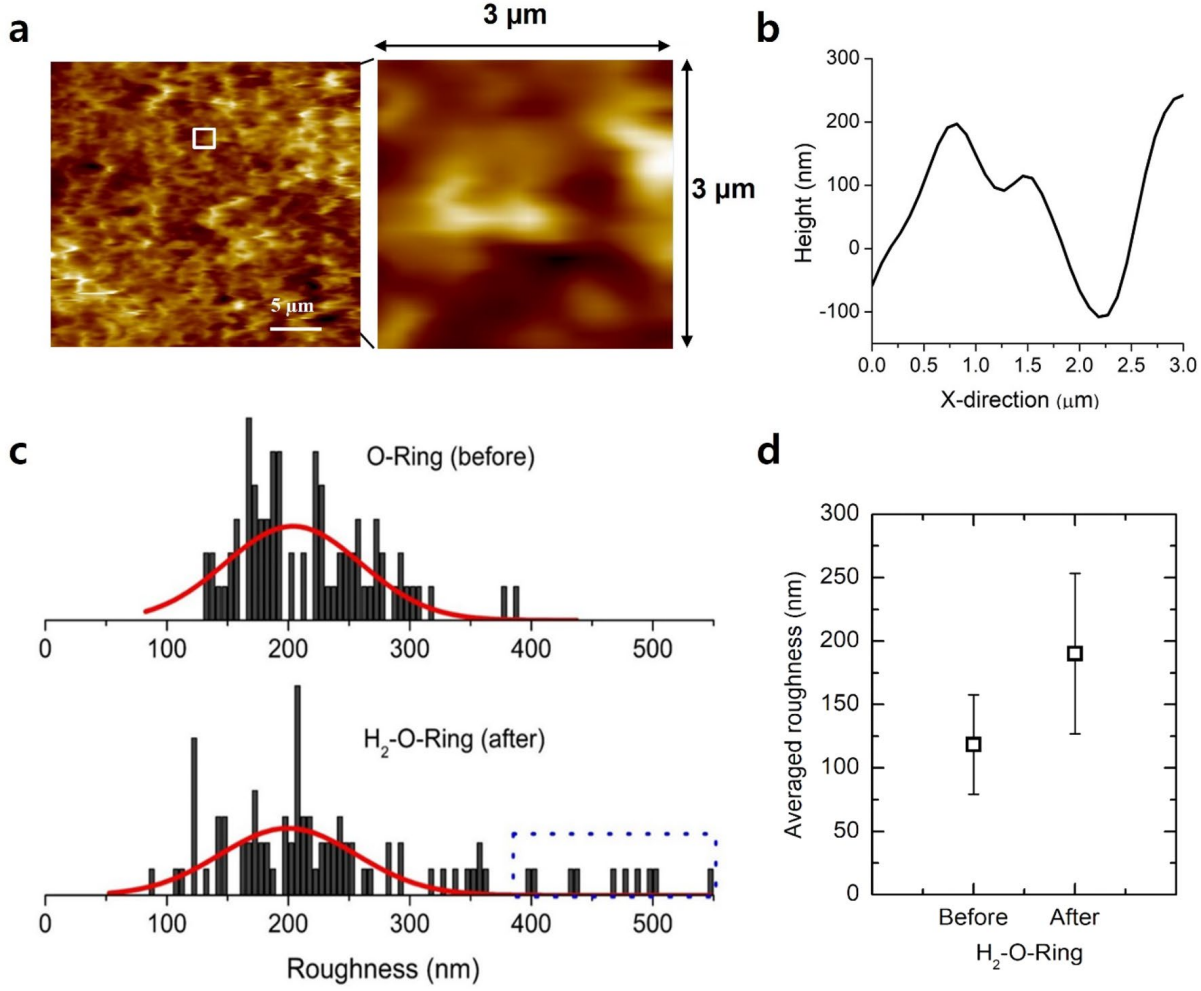
(**a**) A representative illustration showing a sector outlined by a white rectangular box among 100 distinct regions used to calculate surface roughness from the outer surface area. (**b**) Height profiles obtained along the X-direction within the white rectangular box. (**c**) Roughness distributions obtained from the outer surface of the O-ring before (upper) and after (lower) exposure to H_2_ at a pressure of 70 MPa. (**d**) The resulting average roughness before and after exposing the O-ring to 70 MPa of H_2_ gas.

When analysing the surface roughness, the cross-sectional cutting and slice cutting surfaces are not scientifically meaningful due to the artificial effects introduced during the cutting process. However, the outer-radius surface retains the direct effects associated with the hydrogen discharge process, making it highly useful for surface roughness analysis. Therefore, a comparison of the surface roughness before and after hydrogen exposure was conducted solely based on the outer-radius surface. In this comparison, two-dimensional AFM images of the O-ring were first corrected using a plane correction process to account for the cylindrical shape of the O-ring. The average roughness value (S_a_) was then measured within a 3 × 3 μm^2^ area to mitigate errors introduced by the cylindrical shape of the O-ring. The roughness of each group was assessed across more than 100 distinct regions, as depicted in Fig. 7a. The line profile depicts the height variation of the magnified surface image along a white line, from which the surface roughness value is derived (Fig. 7b).

Figure 7c illustrates the roughness distributions of the outer-radius surface before and after exposure to H_2_ at a pressure of 70 MPa. Prior to H_2_ exposure, the roughness exhibited a single Gaussian distribution^40,41^. However, after exposure to H_2_ at a pressure of 70 MPa, a tailing distribution emerged in the high roughness region, as depicted by the blue dashed rectangular box in Fig. 7c. In other words, the low roughness region followed a Gaussian distribution, indicating a random surface roughness associated with random fracturing. In contrast, the high roughness region did not conform to a Gaussian distribution but showed a tailing distribution, suggesting the absence of random fracturing. The averaged roughness also increased by H_2_ exposure (Fig. 7d).

This phenomenon is believed to be related to H_2_ exposure. The increase in roughness primarily results from the expansion of the fracture surface area during crack propagation, leading to increased roughness on the fracture surface. Furthermore, the roughness is broadened after exposure to hydrogen at a pressure of 70 MPa.

**Figure 8:**
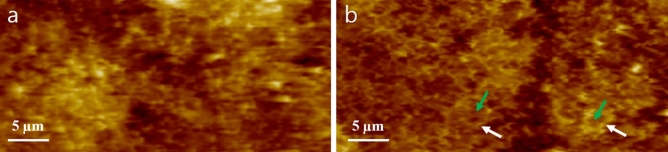
AFM images obtained from the slice cutting surfaces of the O-ring (**a**) before and (**b**) after exposure to H_2_ at a pressure of 70 MPa.

Figure 8 presents a circular morphology on the slice cutting surface. Before exposure to H_2_ (Fig. 8a), the AFM images do not display any circular morphology; they only show bright spots with a gold colour. However, after exposure to H_2_ at a pressure of 70 MPa, numerous circles with a bright gold colour emerge, surrounding dark brown channels (indicated by green arrows in Fig. 8b). These dark brown channels are believed to correspond to pathways for H_2_ gas diffusion in the radial direction of the O-ring, while the bright gold circles (indicated by white arrows in Fig. 8b) mark the periphery of these channels. Interestingly, the channel-like patterns are also directly observable in the AFM images of the cross-sectional cutting surface.

**Figure 9:**
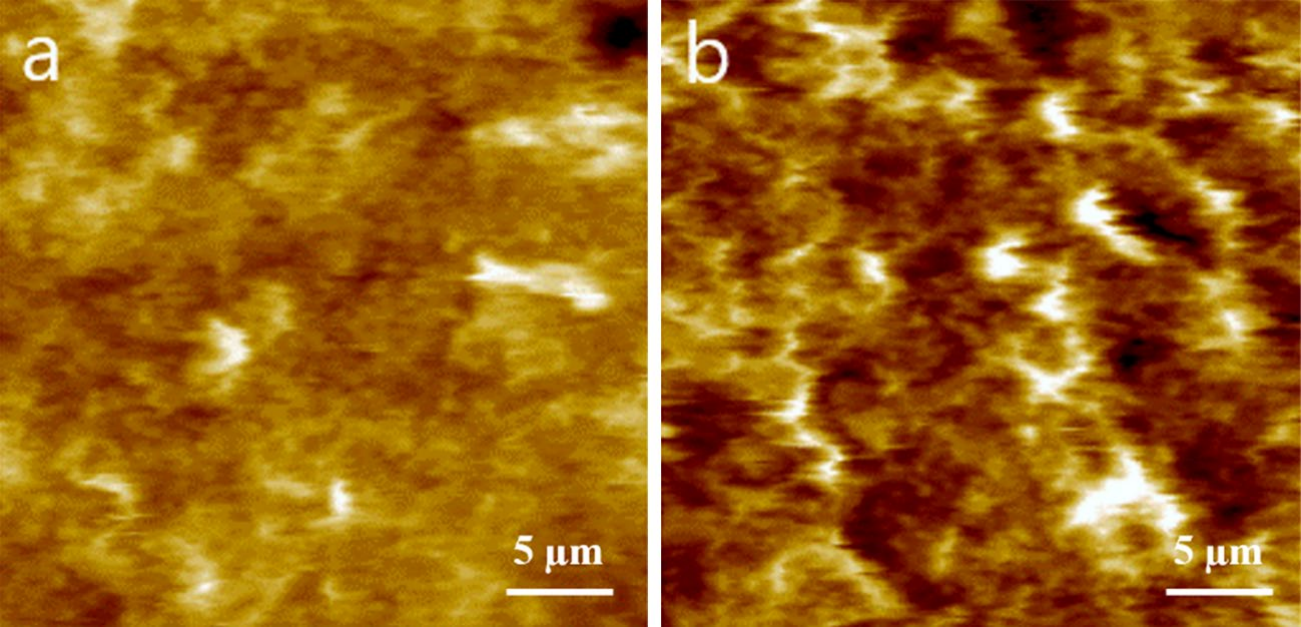
AFM morphologies obtained from the cross-sectional cutting surface of the O-ring (**a**) before and (**b**) after exposure to H_2_ at a pressure of 70 MPa.

As depicted in Fig. 9, no channel-like morphology is present on the cross-sectional cutting surface before exposure to H_2_ (Fig. 9a). However, after exposure to H_2_, a channel-like morphology becomes evident in the AFM image (Fig. 9b), with an average width of approximately 1.9 nm (± 0.76 nm).

**Figure 10:**
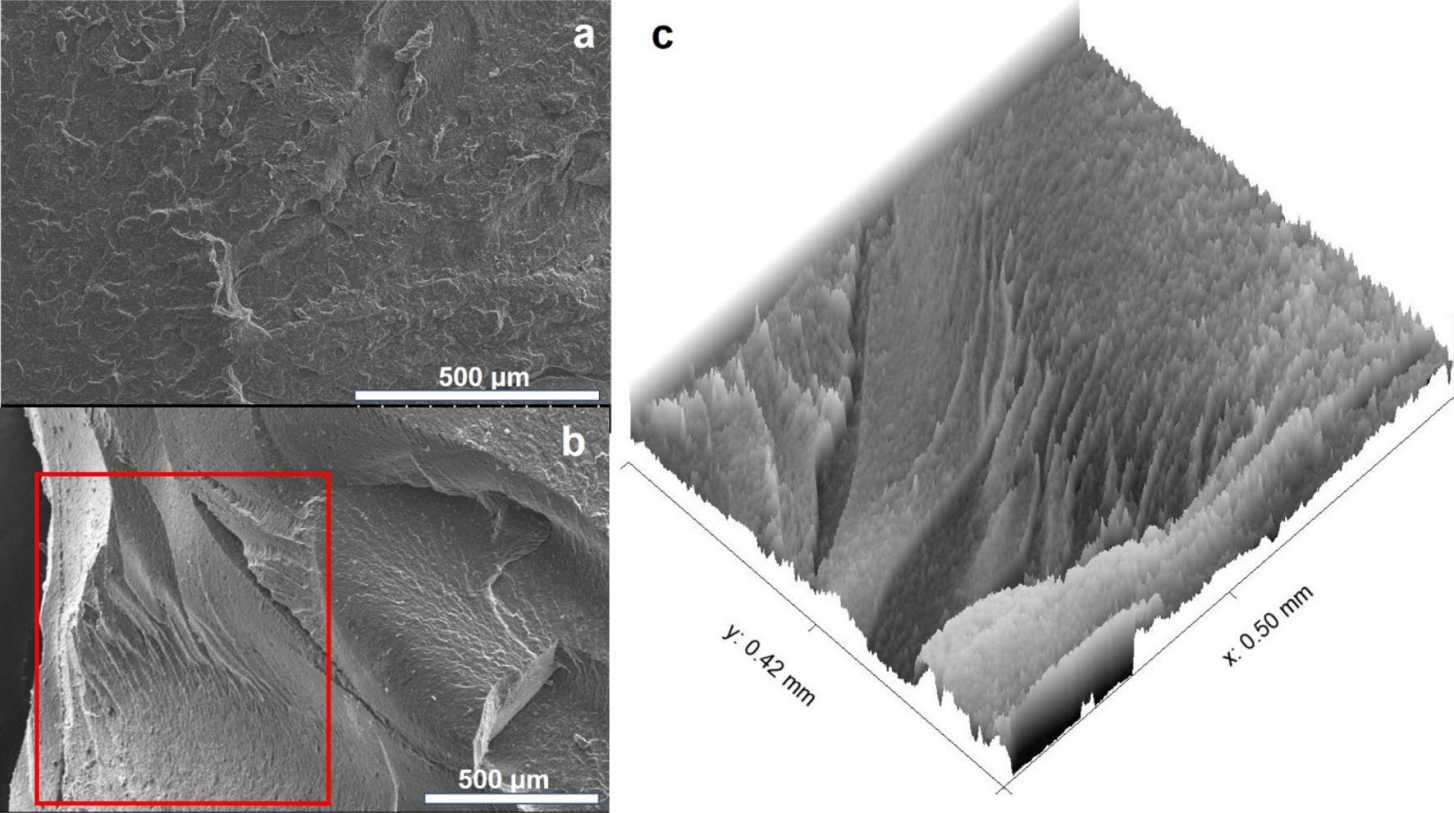
SEM images of the cryo-fracture surface of the O-ring (**a**) before and (**b**) after exposure to H_2_ at a pressure of 70 MPa. (**c**) Enlarged view in 3-dimensional mode of the highlighted area indicated by the red rectangular box.

Further evidence of the channel morphology can be observed in Fig. 10 obtained using SEM. Upon exposure to H_2_, the formation of channel structures becomes apparent. In the SEM image of the cryo-fracture surface of the O-ring without H_2_ exposure (Fig. 10a), no specific directional pattern is observed in the morphology. However, in the SEM image of the O-ring subjected to H_2_ at a pressure of 70 MPa (Fig. 10b), a clear hierarchical development of channel structures is visible on the cryo-fracture surface. This can be observed within the rectangular red box located at the lower left of Fig. 10b. The left and right sides of Fig. 10b represent the inner and outer radial parts of the O-ring cutting plane, respectively. Under the condition of a H_2_ pressure of 70 MPa, the outer radial side of the O-ring is exposed to atmospheric pressure (0.1 MPa), while the left inner radial side of the cryo-fracture surface is subjected to H_2_ at a pressure of 70 MPa. Considering the O-ring thickness of 5.7 mm, the pressure difference per unit thickness can be calculated to be approximately 12 MPa/mm (or 12 GPa/m). The pressure difference between the inner and outer sides of the O-ring is significant, causing expansion of the O-ring. This expansion can lead to tearing due to the tensile stress experienced by the FKM composite, which typically ranges up to approximately 30 MPa.

Furthermore, when the O-ring is constrained by a mechanical force, the blooming phenomenon predominantly occurs in the outer radial direction. As a result, the O-ring undergoes uniaxial directional cavitation. The observed damage patterns, characterized by hierarchically ordered channels, as shown in Fig. 10b,c, do not indicate isotropic cavitation induced by depressurization.

**Figure 11:**
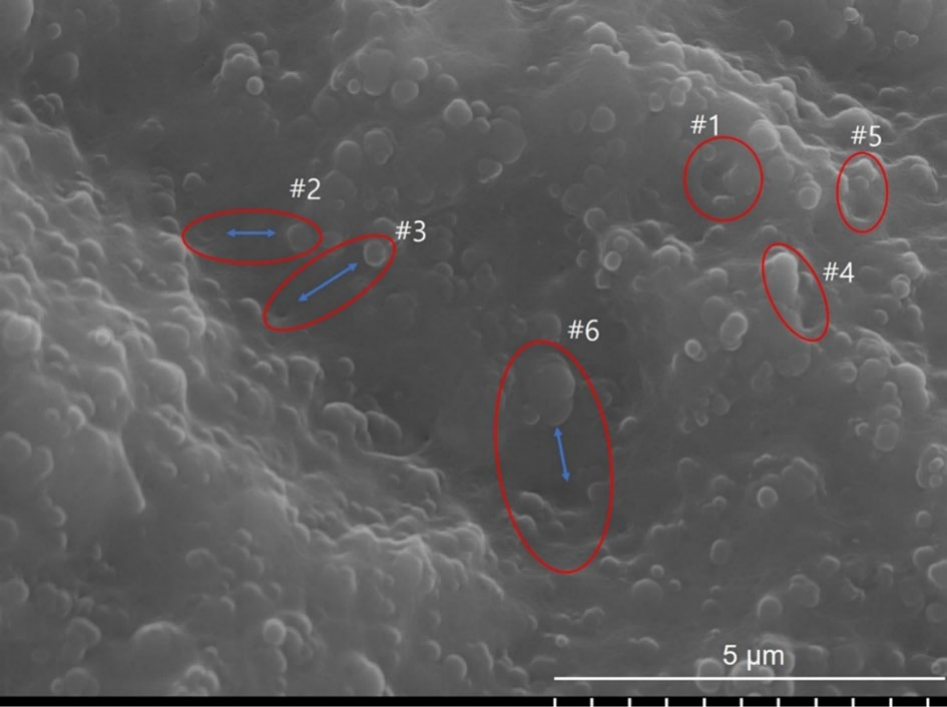
SEM image of the inside of a channel, showing the separation of CB fillers from the rubber matrix. Red circles and ellipses highlight representative examples of filler separation. Bilateral blue arrows indicate the corresponding counterparts of the hollows in the rubber matrix and their corresponding fillers. The symbols "#" and Arabic numbers are used to identify the pairs of rubber matrix hollows and fillers.

Within the channels, another notable phenomenon becomes apparent. Figure 11 presents an SEM image that captures the channels and reveals the presence of small black hollows within the channel interiors and in their surrounding areas. These hollows are marked with red circles (or ellipses) and numbered from #1 to #6. The structures resembling spherical particles observed within the channels represent CB fillers, while the background corresponds to the matrix consisting of rubber molecules. The fillers can be observed in both aggregated and primary forms. Importantly, note that these structures within the channels were not formed as a result of the cryo-fracture process, but rather, they were already present due to the hydrogen depressurization.

The red circles (or ellipses) labelled #1 to #6 represent three distinct types of separated pair structures of the hollows and their corresponding fillers. These separations correspond to the initial stage of filler separation from the rubber matrix (#1), single filler separation (#2, #3 and #4), and separation of filler aggregates (#5, #6)^42^. In the case where separation has just begun (#1), the filler aggregates are observed to be partially detached from the polymer matrix, providing strong evidence of their separation^10^. The filler aggregates partially protrude, creating holes in the rubber matrix where the fillers have been removed. In the case of single filler separation (#2, #3 and #4), the portion of the rubber matrix from which the filler left, indicated by blue arrows, exhibits a smaller hollow structure than the corresponding filler itself. This can be attributed to the elastic restoration of the rubber matrix. However, the filler aggregates (#5, #6) do not exhibit similar behaviour, as they have undergone a wider range of separation, leaving insufficient time for the disrupted structure to restore itself. Specifically, the black hollows and the corresponding CB fillers of #2, #3 and #6 are positioned on the nearly perpendicular opposite sides of the deepest valley. Furthermore, the distances between the separated CB fillers and the rubber matrix inside the channel (#2, #3, #6) are greater than the distances between the separated CB fillers and the rubber matrix outside the channel (#1, #4, #5). This observation strongly suggests that the separation between the fillers and rubber matrix occurred during the formation of the channel. If this observation holds true, then it implies that the CB fillers are not fully embedded within the polymer matrix but rather partially exposed. Consequently, the fillers cannot effectively bind with the polymer matrix, resulting in loss of their intended functionality.

In summary, exposure to hydrogen gas at 70 MPa does not significantly affect the lattice parameters of the O-ring material. However, in the range of micrometres, the formation of a hierarchical channel morphology becomes evident. This channel formation process is accompanied by the separation of fillers from the rubber matrix, leading to mechanical failure.

This phenomenon can be explained by the pressure gradient established between the inner and outer radii of the O-ring under the compression pressure perpendicular to the radius due to clamp locking. Typically, the application and release of hydrogen pressure in a simulated ambient H_2_ pressure environment are carried out under surrounding ambient pressure conditions. This results in the formation of voids uniformly in all directions during the depressurization process.

However, in real operating conditions, the O-ring is mechanically constrained perpendicular to its diameter, leading to a pressure gradient between the inner and outer sides of the O-ring. As a consequence, the voids formed in the O-ring during actual usage encounter difficulties in expanding uniformly. Consequently, the expansion process is more likely to occur in an anisotropic manner along the radial direction, even though the expansion volume remains the same as that observed in isotropic expansion.

## Methods

### O-ring specimen

The O-ring material used in this study was commercially available (VEA1049-12 O-ring P112, PRETECH CO. LTD., Japan). The O-ring specimen (thickness: 5.7 mm) was subjected to two cycles of sealing with high-pressure hydrogen gas at 70 MPa. After this exposure, the O-ring experienced a loss in its sealing performance.

### Powder X-ray diffraction (PXRD)

Powder X-ray diffraction (PXRD) analysis was conducted using a model X’pert Pro X-ray diffractometer (PANalytical Co., Netherlands). The X-ray generator was set to 30 mA and 40 kV, with a receiving slit size of 0.300 mm. The O-ring sample was placed on the goniometer, and a 2θ Bragg angle scan was performed from 10.0100° to 79.9900° with a step size of 0.0200° and a measurement time of 1 s at room temperature.

### Small-angle X-ray scattering (SAXS)

Small-angle X-ray scattering (SAXS) experiments were carried out at the 4C SAXS II beamline of the Pohang Light Source II (PLS II). Monochromatic X-rays with a wavelength (λ) of 0.7560 Å were used. The beam path was maintained under vacuum to minimize air scattering, and the sample-to-detector distance was 4 m. A two-dimensional charge-coupled detector (Mar USA, Inc.) was used, and the samples were directly placed on the sample holder. The two-dimensional scattered intensity was azimuthally averaged to obtain the one-dimensional intensity as a function of q, covering a q range of 0.007 < q (Å^−1^) < 0.14. The raw data were corrected for detector noise, background scattering, and transmission. The experiments were conducted at both room temperature and 80 °C, and the SAXS intensity was not converted to absolute intensity. The temperature was controlled using a Eurotherm, and the heating rate was approximately 10 K/min. For the measurements at 80 °C, the sample was kept at this temperature for two minutes before the measurements were performed. The measurement time was 3 s for room temperature and 2 s for the 80 °C measurements.

The SAXS data were fitted to Eq. (1) in the manuscript, where *R*_*c*_ represents the core radius derived from the solid sphere form factor, and *p*.*d*.*i*. signifies the size distribution of the core radius, as implemented in the Schulz-Zimm distribution function. It's crucial to note that the *R*_*c*_ value was computed under the condition that the background value is not treated as a fitting parameter but is fixed at zero. In fact, the background itself, particularly near the high *q* maximum, exhibits noise, a detail that was evident in our data but not explicitly visible in the log–log plot. Furthermore, it is accurate that the core radius obtained from the fit closely aligns with the maximum high *q*. In response to this, we attempted to concentrate the fit on the low *q* range of the solid sphere form factor, which typically reveals an oscillating pattern in the form factor. The standard deviation for both *R*_*c*_ and *R*_*g*_ was determined as the fit error from the function incorporated into the NIST SANS package. This fit error was cross-verified by fixing one fit parameter while allowing others to vary within the program.

### Field emission-scanning electron microscopy (FE-SEM)

Field emission-scanning electron microscopy (FE-SEM) images were acquired using a Hitachi S-4800 microscope operating at 15 kV and 5.1 μA at room temperature. The imaging mode used was mixed lower–upper mode.

### Atomic force microscopy (AFM)

Atomic force microscopy (AFM) was performed to examine the morphology of the O-ring using a NANO Station II instrument (Surface Imaging Systems, Herzogenrath, Germany). The AFM system consisted of an AFM scanner with dimensions of 92.5 × 92.5 × 6.0 µm^3^ in the x, y, and z directions and a Zeiss optical microscope (Epiplan 500×). A TS-150 active vibration isolation table (S.I.S., Herzogenrath, Germany) was used to reduce the noise signals from vibrations. The surface images were acquired in noncontact mode with a scan resolution of 512 × 512 pixels and a scan speed of 0.7 line/s. The AFM probe used consisted of a force-sensing or -imposing cantilever with a silicon material, a spring constant of 0.2 N/m (± 0.15 N/m), a length of 450 µm (± 10 µm), a width of 30 µm, and a thickness of 2 µm (± 1 µm). The tip used had a radius of 5–6 nm and a height of 14 µm (± 2 µm).

## Data Availability

The datasets generated and/or analyzed during the current study are available from the corresponding author on reasonable request.
